# A homozygous double mutation in *SMN1*: a complicated genetic diagnosis of SMA

**DOI:** 10.1002/mgg3.10

**Published:** 2013-05-30

**Authors:** Susan M Kirwin, Kathy M B Vinette, Iris L Gonzalez, Hind Al Abdulwahed, Nouriya Al-Sannaa, Vicky L Funanage

**Affiliations:** 1 Molecular Diagnostics Laboratory, Nemours/Alfred I. duPont Hospital for ChildrenWilmington, Delaware, 19803; 2 Dhahran Health CenterDhahran, Saudi Arabia

**Keywords:** Capillary electrophoresis, duplication, *SMN* mutations, *SMN1*, spinal muscular atrophy

## Abstract

Spinal muscular atrophy (SMA), the most common autosomal recessive cause of infant death, is typically diagnosed by determination of *SMN1* copy number. Approximately 3–5% of patients with SMA retain at least one copy of the *SMN1* gene carrying pathogenic insertions, deletions, or point mutations. We report a patient with SMA who is homozygous for two mutations carried *in cis*: an 8 bp duplication (c.48_55dupGGATTCCG; p.Val19fs*24) and a point mutation (c.662C>T; p.Pro221Leu). The consanguineous parents carry the same two mutations within one *SMN1* gene copy. We demonstrate that a more accurate diagnosis of the disease is obtained through a novel diagnostic assay and development of a capillary electrophoresis method to determine the copy number of their mutant alleles. This illustrates the complexity of *SMN* mutations and suggests additional testing (gene sequencing) may be appropriate when based on family lines.

## Introduction

Spinal muscular atrophy (SMA) is an autosomal recessive disease characterized by progressive loss of lower motor neurons, resulting in weakness and muscle atrophy (Lefebvre et al. 1995) and is classified based on the severity of symptoms: type I, Werdnig-Hoffman (OMIM 253300), usually presents at <6 months of age; type II (OMIM 253550) onsets typically at <18 months of age; type III (OMIM 253400), Kugelberg-Welander, is late onset, >24 months of age; and type IV (OMIM 271150) is a young adult/adult onset form with muscle weakness and variable progression. Frequency in the Western world is ∼1/6000–1/10,000 live births (Lunn and Wang 2008; Wilson and Ogino 2008), whereas one Saudi study reported as many as 1.93/1000 live births classified as SMA type I (Al Rajeh et al. 1992). A 500 kb inverted duplication in chromosome 5q13 codes for *SMN1* (survival of motor neuron 1) and its paralogue *SMN2* and is subject to frequent deletions, conversions, and mutations. Whereas *SMN1* is the fully functional gene copy, *SMN2* mRNA is inefficiently spliced, with the majority of *SMN2* transcripts lacking exon 7. SMA is predominantly caused by homozygous deletion/conversion of the *SMN1* genes (OMIM 600354). More than 95% of patients inherit no intact copies of *SMN1*, with severity often attenuated by the presence of more than two copies of *SMN2* (Wirth et al. 1999; Feldkötter et al. 2002). The remaining 3–5% of SMA patients retain at least one copy of *SMN1* (Bussaglia et al. 1995; Cuscó et al. 2003; Fraidakis et al. 2012; Qu et al. 2012). In these patients, sequencing has revealed subtle pathogenic mutations in *SMN1*, consisting of base substitutions resulting in amino acid substitutions, termination codons, splice site alterations, and small insertions or deletions resulting in translational frameshifting of the remaining *SMN1* gene transcript (Wirth et al. 1999; Wirth 2000; Ogino and Wilson 2004; Sun et al. 2005). Thus, DNA sequencing is clinically indicated in individuals presenting with SMA symptoms who have only one copy of *SMN1*, although usually is not considered in those with two or more copies of *SMN1*.

**Figure 1:**
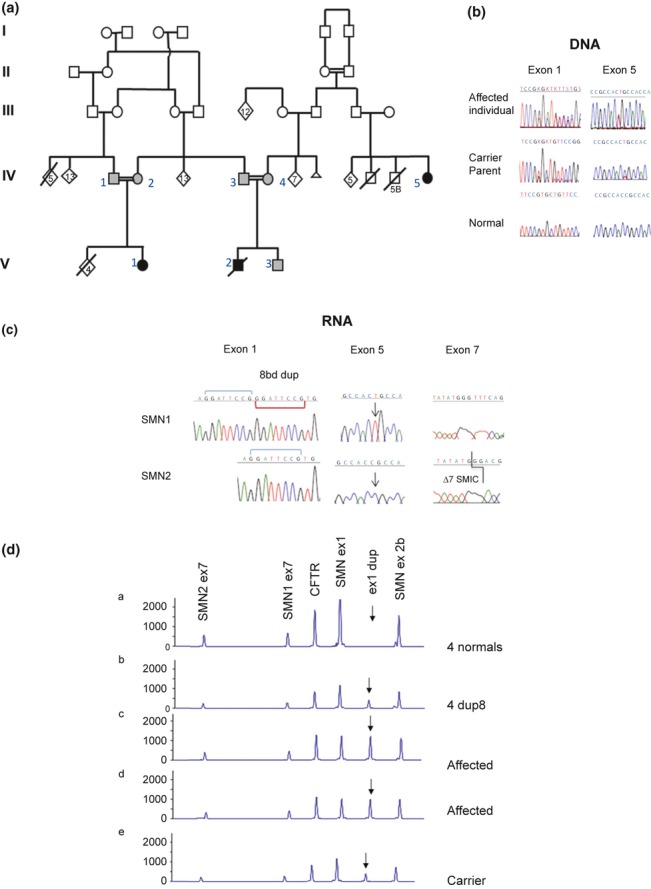
*SMN1* mutations (a) Extended family pedigree. Solid black symbols indicate individuals affected with type I SMA, gray symbols represent carriers of the *SMN1* copy with two mutations, and open symbols indicate individuals for whom no clinical or molecular information is available. (b) DNA sequencing results indicate a frameshift in exon 1 due to the 8 bp duplication. The point mutation in exon 5 is shown as a single heterozygous site; normal control is shown for reference. (c) RT-PCR sequencing chromatograms of exons 1, 5, and 7 of *SMN1* and *SMN2* products in patient V-1. The duplication is only detected when *SMN1*-specific PCR primers are utilized for amplification of exons 1 through 7 from RNA followed by subcloning. Amplification using *SMN2* Δ7-specific primers resulted in the expected normal nucleotides in both exon 1 and exon 5, further confirming that the mutations are located within the *SMN1* genes. (d) CE fragment analysis using fluorescent-based multiplex PCR of the exon 7 region of *SMN1*, *SMN2*, and exon 4 of *CFTR*, with subsequent DraI-digestion to detect *SMN2*. Sizes detected: *SMN2*, 164 bp, and *SMN1*, 187 bp; *CFTR*, 195 bp; *SMN* exon 1 normal, 202 bp; *SMN1* duplicated, 8 bp, 210 bp; *SMN* exon 2b, 220 bp. (a) A pool of four normal controls with two copies of *SMN1* and two copies of *SMN2*; (b) Pool of four carrier (heterozygous) parents each with one copy of a normal *SMN1* exon 1 and one copy with the 8 bp duplication; (c) and (d) Affected patient with two copies of the 8 bp duplication; (e) A carrier parent with one copy of the 8 bp duplication within *SMN1*. CE, capillary electrophoresis; *CFTR,* cystic fibrosis transmembrane regulator; PCR, polymerase chain reaction; RT, reverse transcription; *SMN,* survival of motor neuron.

In this report, we describe a patient with SMA (V-1; Fig. 1a) and several affected family members carrying two *SMN1* copies each with two different *SMN1* mutations. The proband presented at birth with hypotonia, muscle weakness, absent deep tendon reflexes, and respiratory insufficiency, and was clinically diagnosed with SMA type I. A spinal cord magnetic resonance image (MRI) indicated a normal appearance; however, DNA sequencing of the *SMN* genes in this patient revealed two different mutations: an eight-base-pair duplication in exon 1 (c.48_55dupGGATTCCG; p.Val19fs*24) and a separate point mutation within exon 5 (c.662C>T; p.Pro221Leu). Neither mutation has been described previously in the literature or in an available *SMN* database (http://www.dmd.nl). The large extended family pedigree (Fig. 1a) showing muscle disease, infant death, and consanguinity supported the diagnosis in the initial proband with two copies of *SMN1*.

Heterozygous changes in the DNA sequencing chromatograms (Fig. 1b) were deceiving, as the standard *SMN* polymerase chain reaction (PCR) results in the amplification of both *SMN1* and *SMN2* genes. Heterozygous sites cannot be assigned unequivocally to *SMN1* or *SMN2* as the genes differ at only five known sites throughout the intron 6, exon 7, intron 7, and exon 8 regions. To clarify the inheritance of the mutations in this family, we requested parental blood samples and an additional sample on patient V-1 for isolation of RNA. We anticipated that each parent (IV-1 and IV-2) would carry one of the mutations, thus contributing to the SMA phenotype in the daughter.

DNA sequencing analysis for the consanguineous parents revealed that each carries both the exon 1 (p.Val19fs*24) and the exon 5 (p.Pro221Leu) mutations (Fig. 1b). As the parents are healthy, it was predicted that the two mutations either had to be *in cis* on one *SMN1* allele, or one resided in *SMN1* and the other in *SMN2*.

To determine the location of the mutations, we performed reverse-transcription PCR (RT- PCR) on total RNA, followed by cloning and sequencing to separate and distinguish full-length (FL) *SMN1*-derived clones from *SMN2* Δ7 (exon 7–skipped)-derived clones. The cloned parental cDNA sequences supported our hypothesis that each parent produced two types of FL *SMN1* transcripts: one transcript with the two mutations and the other clearly without these mutations (Fig. 1c). Neither of the mutations was found in the *SMN2* Δ7 transcripts (Fig. 1c). Thus, the mutations segregated in the family on the same *SMN1* allele. This verified that both *SMN1* gene copies of patient V-1 carried these two mutations.

We subsequently diagnosed additional affected members of this pedigree. Patient V-2 was referred for *SMN* DNA sequencing after presenting at 3 months with a clinical phenotype of general diffuse weakness and tongue fasciculations and with normal brain MRI results. DNA sequencing and dosage analysis confirmed that his two *SMN1* genes carry both the exon 1 and exon 5 mutations. His healthy parents (IV-3; IV-4) were found to be heterozygous for one mutant *SMN1* allele, carrying both the exon 1 and exon 5 mutations and one normal allele. Prenatal testing for mutation detection was requested several months later when this mother became pregnant. However, the DNA sequences of affected individuals are indistinguishable from one-copy carriers of the mutated allele because of the *SMN2* background amplification, so we developed a quantitative capillary electrophoresis (CE) fragment analysis assay specific to the familial duplication that differentiates between the normal-sized and mutant *SMN* alleles and also provided *SMN1* and *SMN2* copy number. The test amplifies *SMN* exons 1, 2b, and 7, and *CFTR* exon 4 as an internal control, allowing simultaneous determination of *SMN1* and *SMN2* gene copy numbers by quantitation of the distinguishable exon 7 region, with concomitant copy numbers of mutant and normal-sized *SMN* exon 1 regions. It distinguishes one-copy carriers of the allele with the 8 bp duplication from two-copy carriers of the mutations in potentially affected individuals. Figure 1d shows the size-based separation of the normal-sized exon 1 region and the 8 bp duplication size variant so their peak areas could be measured. Normal individuals did not display the duplicated 8 bp fragment (Fig. 1d) that was detected in the four parents (Fig. 1d) and the affected children (Fig. 1d). Analysis of area of the peaks (Table 1) showed that the affected children carry twice as many copies of exon 1 with the 8 bp duplication as the carrier parents. Results of columns 1 and 2 indicate two copies of *SMN1* and *SMN2* for all individuals tested (ratio equals 1). For the exon 1 region, there are two measurements: analyses for the exon 1 duplicated region and for the normal-sized exon 1. Columns 3 and 4 show unaffected individuals (4NLS) having only normal exon 1 (both from *SMN1* and *SMN2*); affected individuals have twice as many copies of the duplicated region compared with carriers and only half the number of copies of the normal-sized exon 1 region compared with controls. The *SMN1* and *SMN2* copy number at the exon 7 region is measured after *DraI* enzyme digest, which allows for the distinction of *SMN1* from *SMN2*. All individuals tested show two copies each of exon 7 *SMN1* and *SMN2*.

**Table 1:** Determination of 8 bp duplication copy number by fragment analysis assay

Sample	Ratio *SMN1* exon 7/*CFTR*	Ratio *SMN2* exon 7/*CFTR*	Ratio *SMN* exon 1 dup8/*CFTR*	Ratio *SMN* exon 1 normal/*CFTR*	Copy number exon 1 normal (*SMN1* + *SMN2*)	Copy number exon 1 8 bp duplication (*SMN1*)	Copy numbers ratio exon 7 *SMN1* to *SMN2*
4NLS	1.00	1.00	n/a	1.00	4	0	2:2
Four carriers	1.00	1.00	1.00	1.00	3	1	2:2
V-1	0.98	0.91	1.92*	0.59	2	2	2:2
V-2	1.06	0.98	1.85*	0.59	2	2	2:2
Carrier parent	0.90	0.81	0.95	0.88	3	1	2:2

A pool of four normal copy number control DNAs (2 copy *SMN1* exon 7 and 2 copy *SMN2* exon 7) comprise the 4NLS sample. A pool of four carrier parents is used to standardize the duplicated eight-base-pair region of exon 1. All other calculations are based on the ratios of the area under the peak of the gene of interest to *CFTR* and normalized to the 4 NLS. *CFTR,* cystic fibrosis transmembrane regulator; V, related individual; NLS, unaffected individual; *SMN,* survival of motor neuron.

* Results of V-1 and V-2 indicate they carry two copies of the duplicated 8 bp exon 1 region with values approaching 2.0 compared to controls.

The CE data confirmed and validated the results of the RT-PCR cloning and sequencing of the original proband and her parents' mRNA, indicating that V-1 and V-2 have inherited only the *SMN1* allele with the 8 bp duplication. The individual V-3 (a prenatal DNA sample) was found to be a carrier of one allele with the *SMN1* mutations and one normal *SMN1* allele (Fig. 1d).

A fourth related individual (IV-5) with a clinical history of severe hypotonia, ventilator dependency, an electromyogram (EMG) consistent with axonal disease, and a family history of infant death was referred for *SMN* sequencing testing at age 11 years. Utilizing the family-specific fragment analysis assay and *SMN* gene sequencing, we confirmed that this affected individual was homozygous for the same *SMN1* mutations as previously identified in V-1 and V-2.

The phenotype of the affected patients must be caused by the null mutation in exon 1; in this case, the exon 5 change would not be expressed at the protein level, and its effect on phenotype would be negligible. It is difficult to predict the effect of the p.Pro221Leu change by itself: the pathogenicity prediction programs SIFT (http://www.sift.jcvi.org) and PANTHER (http://www.pantherdb.org) classify leucine as not tolerated, but other programs yield inconclusive results. *SMN* exons 4, 5, and 6 contain extended runs of prolines, and our alignment of *SMN* proteins shows variable numbers of prolines in primate and prosimian exon 5. There is a leucine in the middle of the *Pan troglodytes* proline sequence, but not in *Pan paniscus*. These primate sequences suggest that leucine may be tolerated among the prolines.

It was imperative to investigate how these mutations were inherited through the family lines so the family could be properly counseled. Consanguineous marriages are customary throughout the Middle East; statistics reported are as low as 34% and as high as 80%, with regional variations (al Rajeh et al. 1993; Al Rajeh et al. 1998). The prevalence and genetic effects of consanguinity have been addressed previously regarding the different frequencies of autosomal dominant and autosomal recessive conditions (including SMA) in the Middle East and Asia (Salih et al. 1996; Majumdar et al. 2005; Al Jumah et al. 2007; Tadmouri et al. 2009). Founder effects within large extended families may be responsible for a higher incidence of autosomal recessive diseases and should be considered when counseling a family for SMA and other autosomal recessive disorders (Modell and Darr 2002; Al Jumah et al. 2007).

Although homozygosity for *SMN1* subtle mutations has been reported in consanguineous families (Bussaglia et al. 1995; Cuscó et al. 2003), this is the first report of a patient with SMA homozygous for two subtle mutations *in cis*. Clarification of the location of these mutations in the *SMN1* genes was complicated by parental consanguinity; it required *SMN* exon 7 deletion assay, *SMN* DNA sequencing, analysis of parental DNAs, RT-PCR, cloning, and sequencing of the patient and parental mRNAs to unequivocally separate the FL message from the Δ7 message and also required development of a CE test designed specifically for this mutation. Here, our molecular genetic findings followed the inheritance of two mutations on each *SMN1* allele in the affected individuals. As this protocol has been validated in this multigeneration family, it would be sufficient to use the CE assay and provide carrier status in the future with the knowledge that this test provides *SMN1/SMN2* quantitation for copy numbers. This CE assay will be equally informative if the more common *SMN1* deletion chromosome is also present in the extended family. Compound heterozygous mutations consisting of the typical *SMN1* deletion/conversion and a second allele harboring a subtle mutation account for some 5% of SMA cases. Our results show that a search for subtle mutations and their precise location may be indicated in patients with SMA who have two *SMN1* alleles.

## Accession Numbers

The accession number for the reference sequence reported in this study is NM_000344.3, *SMN1*.
